# Microbial Changes and Host Response in F344 Rat Colon Depending on Sex and Age Following a High-Fat Diet

**DOI:** 10.3389/fmicb.2018.02236

**Published:** 2018-09-21

**Authors:** Sun Min Lee, Nayoung Kim, Hyuk Yoon, Ryoung Hee Nam, Dong Ho Lee

**Affiliations:** ^1^Department of Internal Medicine, Seoul National University Bundang Hospital, Seongnam, South Korea; ^2^Department of Internal Medicine and Liver Research Institute, Seoul National University College of Medicine, Seoul, South Korea; ^3^Tumor Microenvironment Global Core Research Center, College of Pharmacy, Seoul National University, Seoul, South Korea

**Keywords:** aging, high-fat diet, gut microbiota, rats, Ki67

## Abstract

Gut microbiota, an important component that affects host health, change rapidly and directly in response to altered diet composition. Recently, the role of diet–microbiome interaction on the development of colon cancer has been the focus of interest. Colon cancer occurs more frequently in an aged population, and in males. However, the effect of dietary changes on the gut microbiome has been studied mainly in young males, even though it may vary with age and sex. The aim of this study was to investigate microbial changes and host response in the colons of male and female 6-week-old (young) and 2-year-old (old) Fisher-344 rats exposed to a high-fat diet (HFD). Our results showed that exposure to HFD for 8 weeks decreased the species richness of microbiota (Chao1) and increased *Firmicutes*/*Bacteroidetes* ratio only in aged rats, and not in young rats. Sex differences underlying the alteration by HFD in the gut microbiome were observed in the microbiome of aged rats. For instance, the abundance ratio of *Akkermansia muciniphila* and *Desulfovibrio* spp. increased in response to HFD in young rats and female aged rats, but not in male aged rats. Histological inflammation and cell proliferation of colon mucosa (indexed by Ki67) were significantly increased by HFD even in young rats; aged rats showed significantly higher cell proliferation in the HFD group than in the control. The HFD-induced decrease of species richness and the increase in specific species (*Desulfovibrio* spp. and *Clostridium lavalense*), which produce carcinogenic compounds such as H_2_S and *N*-nitroso compounds, were significantly correlated with Ki67 index. In colon mucosa, the concentration of myeloperoxidase was increased by HFD only in males, and not in females. In conclusion, the results suggest a link between HFD-induced gut dysbiosis (particularly the low species richness and high abundance ratios of *Desulfovibrio* spp. and *C. lavalense*) and cell proliferation of colon mucosa (indicated by Ki67 IHC). In addition, sex differences influence the response of gut microbiome to HFD particularly in old age. Such sex differences in the gut microbiota might be related to sex differences in inflammation in the colon mucosa.

## Introduction

### Gut Microbial Changes Occurring in Rats of Different Sex and Age Following a High-Fat Diet

Microbiota composition changes with age (Bischoff, 2016) and gut dysbiosis influences aged guts by promoting gut permeability and inflammation (Thevaranjan et al., 2017). In addition, multiple species have been identified as playing a role in the adiposity and immune system of the host (Faith et al., 2014).

Recent studies have reported sex differences in gut microbiota composition with varying results (Lay et al., 2005; Mueller et al., 2006; Li et al., 2008; Kovacs et al., 2011; Bolnick et al., 2014; Shastri et al., 2015; Org et al., 2016). Some of these have suggested that sex differences have insignificant or no impact on gut microbiota in mice and humans (Lay et al., 2005; Kovacs et al., 2011). On the other hand, an analysis of gut microbiota by FISH in European study populations showed gender-based effects in the *Bacteroides*–*Prevotella* group (Mueller et al., 2006). In 8-week-old mice of 89 different strains, the role of sex differences in gut microbiota composition was demonstrated by grouping microbial communities based on the effects of gonadectomy and hormone treatment (Org et al., 2016). Furthermore, the effects of dietary change on gut microbiota were sex-dependent in fish and mice aged 9 months and 8 weeks, respectively (Bolnick et al., 2014).

### Host Response in Colon Occurring in Rats of Different Sex and Age Following a High-Fat Diet

Old age is a strong risk factor for colon cancer, with 82.4% of diagnoses occurring at 55 years of age or older and 62% occurring at 65 years of age or older, according to the age distribution of incidence from 2010 to 2014 reported by the SEER Program of the National Cancer Institute (Howlader et al., 2017). Furthermore, the mechanism of constipation, a common feature of aged colons, may occur by colonic dysmotility through fat deposition in tunica muscularis of the ascending colon and decrease in the number of interstitial cells of Cajal and neuronal nitric oxide synthase (nNOS)-immunoreactive cells in rat colons (Jo et al., 2014).

In addition, sex differences play an important role in the screening, prevention, and treatment of various diseases including colorectal cancer (Kim et al., 2015). Interestingly, sex differences exist in the induction of the pro-inflammatory and pro-tumorigenic states (Kim et al., 2015). During colon inflammation, immune responses differ according to sex, impacted by both gene and hormone levels (Klein and Flanagan, 2016). Previous studies have reported protective effects of estrogen in dextran sodium sulfate-induced colitis, as estrogen partially protected female mice against colitis (Babickova et al., 2015). Colon cancer incidents tend to be higher in males compared to females in both humans and mice (Lee et al., 2016). Testosterone has been thought to promote colonic adenomagenesis, which may explain the higher incidence of colon cancer in men (Amos-Landgraf et al., 2014; Lee et al., 2016). Colorectal cancer, which is more often associated with the proximal colon in women than in men, is related to the more aggressive form of neoplasia than distal colon cancer (Pal and Hurria, 2010; Hansen and Jess, 2012).

These sex-dependent differences have been presumed to result from differences in the concentration of sex hormones, expression of genes on X and Y chromosomes, and percentage of body fat (Regitz-Zagrosek, 2013).

### Association Between the Gut Microbiota and Colon Mucosa

Another mechanism of aging-related colorectal diseases is associated with gut microbiota, which is closely related to the health and senescence of the host (Claesson et al., 2012). When germ-free mice were colonized with the gut microbiota from the mice model of inflammation-associated colon cancer, they revealed more and larger tumors (Zackular et al., 2013). The effect of the microbiota was confirmed by treatment of antibiotics, which dramatically decreased both the number and size of tumors (Zackular et al., 2013). In addition, the baseline structures of microbiome in mice were strongly associated with the susceptibility to colorectal tumorigenesis (Baxter et al., 2014). Taxa that were most strongly correlated with higher tumor burden were some species within *Bacteroides*, *Parabacteroides*, *Alistipes*, and *Akkermansia* (Baxter et al., 2014).

Dietary composition and diversity, a major factor that determines the composition of gut microbiota (Claesson et al., 2012), induces predictable shifts in human microbiota, which affects the immune and metabolic parameters in the host (Singh et al., 2017). High-fat diet (HFD) in particular alters the gut microbial composition independent of obesity (Hildebrandt et al., 2009). HFD interacts with gut bacteria to induce intestinal inflammation, through stimulation of pro-inflammatory signaling (Ding et al., 2010). The interactions between HFD and gut microbiota activate nuclear factor (NF)-κB pathway in small intestine, which in turn may upregulate the expression of a downstream enzyme, cyclooxygenase 2 (COX2) (Ding et al., 2010; Shi et al., 2015). The structure of gut microbiota influences on the susceptibility to colonic tumorigenesis through alterations in the production of short-chain fatty acids (SCFA), the induction of inflammation, and mucin degradation (Zackular et al., 2013).

Tumorigenesis in the colon begins with the development of a normal epithelium to a state of hyperproliferative epithelium (hyperplasia) (Brennan and Garrett, 2016). This leads to dysplasia of the epithelium, which has the potential to become a nonmalignant adenoma. In response to stimulation of tumor microenvironment, the adenomas can invade to submucosa and become adenocarcinoma. The characteristics of proneoplastic inflammatory environment, by genetic or bacterial stimuli or cytokines, include the NF-κB activation (Karin and Greten, 2005) and the inflammasome activation (Elinav et al., 2013), which are related to the expression levels of COX2 and caspase-1, the factors measured in this study. Myeloperoxidase (MPO) can generate oxidants that induce DNA damage and mutagenesis (VanderVeen et al., 2001). High MPO activity correlates with the severity of chemically induced colitis, and directly with the colon tumor development (Vowinkel et al., 2004; Roncucci et al., 2008).

### Links Between Aging, Sex, Diet, and Colon Cancer

Despite these findings, no study has yet evaluated both age- and sex-specific variations in gut microbiota for application in colorectal diseases, and particularly not a metagenome study. Taken together, we hypothesized that aging and sex differences are important factors, which influence gut microbiota, as well as HFD-induced changes in microbiota and host responses. From this background, the aim of this study is to investigate microbial changes and host response in colon occurring in rats of different sex and age following a HFD. As it is difficult to control for multiple factors when conducting human studies, our team used an F344 rat model of aging (Jo et al., 2014).

## Materials and Methods

### Animals and Diets

Specific-pathogen-free (SPF) F344/NSIc rats [6-week-old (young age) and 2-year-old (old age) rats] of either sex were used (Orient, Seoul, Korea) (Jo et al., 2014; Lee et al., 2017). The rats were bred under SPF conditions at 23°C and under 12:12-h light–dark cycles. They were provided with unrestricted access to food and water. Rats were divided into groups and were fed *ad libitum* with two different commercial diets: chow diet and HFD (chow: 3.85 kcal/g; HFD: 5.24 kcal/g, 60% kcal from fat, Research Diets, Inc., New Brunswick, NJ, United States). The composition of each diet is shown in **Supplementary Table S1**, provided by Research Diets, Inc. (New Brunswick, NJ, United States). HFD group rats were fed fewer carbohydrates and more lards than control diet-fed rats. Consequently, there were eight groups depending on the different ages, sexes, and diets: Group 1, 6-week-old male control (6w.M.C *n* = 6); Group 2, 6-week-old male HFD (6w.M.HF, *n* = 6); Group 3, 6-week-old female control (6w.F.C, *n* = 6); Group 4, 6-week-old female HFD (6w.M.HF, *n* = 6); Group 5, 2-year-old male control (2yr.M.C, *n* = 4); Group 6, 2-year-old male HFD (2yr.M.HF, *n* = 4); Group 7, 2-year-old female control (2yr.F.C *n* = 5); and Group 8, 2-year-old female HFD (2yr.F.HF *n* = 5). The differing numbers of rats in each group resulted from the natural death of old rats in the middle of the experiment due to senescence. During the 8 weeks of feeding chow or HFD, the food intake and body weight of each rat were measured weekly. After the feeding period, fecal samples and tissue specimens were collected for metagenome sequencing and histological and molecular analysis. Terminal anesthesia was then performed via inhalation of carbon dioxide. This study was carried out in accordance with the recommendations of the Guide for the Care and Use of Laboratory Animals of South Korea. The protocol was approved by the Institutional Animal Care and Use Committee (IACUC) of Seoul National University Bundang Hospital (Permission No. BA1506-178/ 027-01).

### Fecal Sample Collection and Metagenome Sequencing

Feces were collected immediately after defecation, after rats had been fed a control diet or a HFD for 8 weeks. All fecal samples were immediately frozen in liquid nitrogen and stored in -80°C. Genomic DNA was extracted from the fecal samples using QIAamp stool DNA extraction kits (Qiagen, Valencia, CA, United States) following the manufacturer’s recommendations.

Using the extracted genomic DNA, the V3-V4 region of 16S rRNA was amplified with PCR. The primers were 341F (5′-TCGTCGGCAGCGTCAGATGTGTATAAGAGACAG CCTACGGGNGGCWGCAG-3′) and 805R (5′-GTCTCGTGGGCTCGG AGATGTGTATAAGAGACAGGACTACHVGGGTATCTAATCC-3′). PCR products were confirmed with electrophoresis and purified using QIAquick PCR purification kit (Qiagen, Valencia, CA, United States). Samples were shipped to Macrogen Inc. (Seoul, South Korea) for quality control assessment to confirm DNA integrity. Metagenome sequencing was performed using the Illumina MiSeq platform.

The reads were assembled using a program, Fast Length Adjustment of SHort reads (FLASH). The quality of sequencing was evaluated according to the number of total bases and read counts, N percentage (proportion of the number of N bases in total bases), GC percentage, and the percentage of bases with quality scores above Q20 and Q30 (**Supplementary Table S2**). Using CD-HIT-DUP, short reads were then filtered out and extra-long tails were trimmed. Next, filtered reads were clustered at 100% identity, and chimeric reads were identified. Secondary clusters were then recruited into primary clusters. Noise sequences in clusters of sizes below 3 were removed. The remaining representative reads from non-chimeric clusters were clustered using a greedy algorithm into OTUs at a user-specified OTU cutoff (97% ID at species level). Taxonomic assignment and diversity analysis were performed by QIIME. Representative sequences were used from each OTU to assign taxonomy.

To visualize the sample differences, principal coordinate analysis (PCoA) was performed with unweighted UniFrac (Lozupone and Knight, 2005). PCoA plots were generated using QIIME’s “make_2d_plots.py” script. The clustering of samples was explained with the principal coordinate (PC) values. The Unweighted Pair Group Method with Arithmetic mean (UPGMA) tree was created using the “upgma_cluster.py” script of QIIME. The UPGMA clustering was performed with the result file from the “beta_diversity.py” script. We extracted a species list of *p*-value < 0.05 based on the results of statistical analysis using a Mann–Whitney *U*-test comparing the OTU abundance ratio (%) between the control and HFD samples at each age and sex, and also created principal component analysis (PCA) biplots at the phylum level. This list of species generated for PCA analysis is shown in **Supplementary DataSheet S1**. PCA biplots were generated using the R package (version 3.1.2) function “prcomp.” Alpha diversity (Chao1 index) was examined using QIIME’s “alpha_diversity.py” script. A rarefaction curve of Chao1 index was generated using the “alpha_rarefaction.py” script of QIIME. Taxonomic summary bar charts were created for the OTU abundance ratio (%) at the phylum level using GraphPad Prism (version 5.01).

### Linear Discriminant Analysis (LDA) Effect Size (LEfSe) Analysis

Linear discriminant analysis (LDA) effect size (LEfSe) analysis^1^ was conducted in order to determine the taxonomic compositions that were significantly changed by diet (Segata et al., 2011). The conditions of LEfSe analysis were: (1) an alpha value for the factorial Kruskal–Wallis test between control and HFD less than 0.05; (2) an alpha value for the pairwise Wilcoxon test among the taxonomic compositions less than 0.05; (3) a threshold of the logarithmic LDA score for discriminative features less than 2.0; and (4) multi-class analysis set as all-against-all.

### Taxonomy Abundance Ratio

The OTU abundance ratios (%) were analyzed at different taxonomic levels, and compared according to age, sex, and diet. The abundance ratios (%) at the level of phylum, class, order, family, genus, and species are shown in **Supplementary DataSheet S2**. Bar graphs of OTU abundance ratio (%) at the species level were created using GraphPad Prism (version 5.01).

### Hematoxylin–Eosin Staining

To evaluate the levels of fat accumulation in the tunica muscularis and inflammation in the colonic mucosa, histological analyses of the proximal colons were performed. For tissue preparation, 1 cm was eliminated from each of the cecum and anus, and 1 cm of the proximal part of the colon was collected. The sample was prepared by fixation in 10% buffered formalin. The tissue specimens embedded in paraffin blocks were cut perpendicularly to the lumen into 4-μm-thick sections and stained with hematoxylin and eosin (H&E). Three H&E-stained slides per rat (in each group, *n* = 4 or *n* = 5 or 6) and four fields per slide were selected at random. The areas of fat tissue and total smooth muscle were quantified using the ImagePro Plus analysis system (Media Cybernetics, Inc., San Diego, CA, United States). The fat proportion was described as the percentage area of fat to that of total smooth muscle (Jo et al., 2014; Lee et al., 2017). Histological scoring was performed by an experimenter who was blinded to the identities of the samples. Colonic epithelial damage and infiltration with inflammatory cells were assigned scores as previously described (Katakura et al., 2005). Briefly, colonic epithelial damage was scored as 0 for normal, 1 for hyperproliferation, irregular crypts, and goblet cell loss, 2 for mild-to-moderate crypt loss (10–50%), 3 for severe crypt loss (50–90%), 4 for complete crypt loss and intact surface epithelium, 5 for small- to medium-sized ulcer (<10 crypt widths), and 6 for large ulcer (≥10 crypt widths). Inflammatory cell infiltration was also scored for mucosa (0 = normal, 1 = mild, 2 = modest, 3 = severe), submucosa (0 = normal, 1 = mild to modest, 2 = severe), and muscle/serosa (0 = normal, 1 = moderate to severe). The sum of the scores of colonic epithelial damage and inflammatory cell infiltration was calculated for each slide (three slides per rat) so as to generate a score of 0–12. The average score of the three slides was used as the score for each rat.

### Immunohistochemical Analysis

Immunohistochemical (IHC) analysis of Ki67 (antigen identified by monoclonal antibody Ki 67), a proliferation marker, was performed. Four fields per section and three sections per rat were analyzed. Tissue sections were treated with 3% hydrogen peroxide and nonspecific binding sites were blocked. The blocking agent was “ultraView Universal DAB Inhibitor,” which contained 3% hydrogen peroxide solution. The sections were incubated with anti-Ki67 antibodies (1:1000 dilution, ab15580, Abcam, Cambridge, MA, United States). The labeled secondary antibodies were used: “ultraView Universal HRP Multimer,” which contained cocktail of HRP-labeled antibodies. An automatic immunostainer (BenchMark XT; Ventana Medical Systems, Inc., Tucson, AZ, United States) and UltraView Universial DAB detection kit (Ventana Medical Systems, Inc., Tucson, AZ, United States) were used for immunostaining. We calculated the proliferation index through dividing the number of Ki67-stained cells by the number of total cells within all crypts (Dermadi et al., 2017).

### Enzyme-Linked Immunosorbent Assay and Western Blot

The scraped proximal colonic mucosa samples were homogenized in lysis buffer and centrifuged. The lysis buffer was composed of radio immunoprecipitation assay (RIPA) buffer, proteinase inhibitor, and phosphatase inhibitor. The supernatant was used for analysis.

Myeloperoxidase concentration in the proximal colonic mucosa was detected through spectrophotometry using an MPO enzyme-linked immunosorbent assay (ELISA) kit (HyCult Biotech, Uden, Netherlands) according to the manufacturer’s instructions.

The protein samples extracted for MPO assay were also used for Western blot. Briefly, for Western blotting analysis, proteins were separated by SDS–PAGE and transferred to PVDF membranes. All procedures were conducted in Tris buffer (40 mM, pH 7.55) containing 0.3 M of NaCl and 0.5% Tween 20. Membranes were blocked with dried milk (5% wt/vol) or bovine serum albumin (3% wt/vol) and blotted with primary antibody followed by a secondary antibody. Anti-COX2 antibody (Cayman 160126), anti-caspase-1 antibody (Santa Cruz sc-56036), anti-cyclin D1 antibody (BD Biosciences 556470), and anti-β-actin antibody (Santa Cruz sc-47778) were used as primary antibodies.

### Statistical Analysis

The body weights, species richness (Chao1), *Firmicutes/Bacteroidetes* ratios, abundance ratios, histology data, and measured values of ELISA and Western blots were compared through a Kruskal–Wallis test, followed by Mann–Whitney *U*-test with Holm–Bonferroni correction. Correlation and regression analysis of the microbiome and histological data were conducted using Spearman’s rank correlation coefficient (also known as Spearman’s rho) and linear regression. These analyses were performed using SPSS version 18 (IBM Corp, Armonk, NY, United States), and *p <* 0.05 was considered to indicate a statistically significant difference.

### Accession Number

The raw unprocessed 16S rRNA gene sequence, which was generated by our study was deposited in the NCBI Sequence Read Archive Database (GEO accession number GSE104184)^2^.

## Results and Discussion

In the present study, the number of animals on each diet per age and sex was only 4–6. This limits the interpretation of our results, owing to the insensitivity in statistics, and the possible loss of stability in PCoA.

### HFD Exposure and Subsequent Changes in Body Weight

**FIGURE 1:**
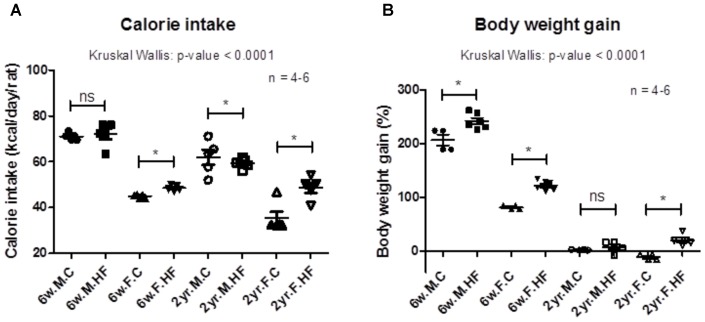
Calorie intake **(A)** and changes in body weight **(B)** during the 8-week feeding of control or high-fat diet (6-week-old rats, *n* = 6; 2-year-old male rats, *n* = 4; 2-year-old female rats, *n* = 5). Data are expressed as mean and SEM; *p*-values to Kruskal–Wallis test is designated on the figure; ^∗^*p* < 0.05 according to Mann–Whitney *U*-test with Holm–Bonferroni correction; M, male; F, female; HF, high-fat diet.

Daily food intake, adjusted as calorie intake, was compared between the control and HFD groups (**Figure 1A**). In female rats, the calorie intake was significantly higher in the HFD groups than in control groups; on the other hand, the calorie intake was not significantly changed by HFD in 6-week-old males, and it was decreased in 2-year-old male rats (*p* < 0.001 to Kruskal–Wallis test; 6-week-old male: *p* = 0.246; 6-week-old female: *p* = 0.009; 2-year-old male: *p* = 0.021; 2-year-old female: *p* = 0.016).

Body weight gain (%) during the 8 weeks of HFD feeding was compared between the control and HFD groups (**Figure 1B**). The weight gain of 6-week-old rats and 2-year-old female rats was significantly higher in HFD-fed rats than in rats fed with control diet; by contrast, it was not increased by HFD in 2-year-old male rats (*p* < 0.001 to Kruskal–Wallis test; 6-week-old male: *p* = 0.010; 6-week-old female: *p* = 0.010; 2-year-old male: *p* = 0.243; 2-year-old female: *p* = 0.014). **Supplementary Figure S1** shows the growth curve of rats during the feeding period. During HFD feeding, the body weight of 6-week-old male rats showed significant increase with HFD at 4th, 5th, and 8th week (at 8th week, *p* = 0.010) (**Figure 1B**). The weights of 6-week-old and 2-year-old female rats also increased with HFD (at 8th week, 6-week old: *p* = 0.010; 2-year old: *p* = 0.014) (**Figure 1B**). However, it was not increased in 2-year-old male rats (at 8th week, *p* = 0.559).

**FIGURE 2:**
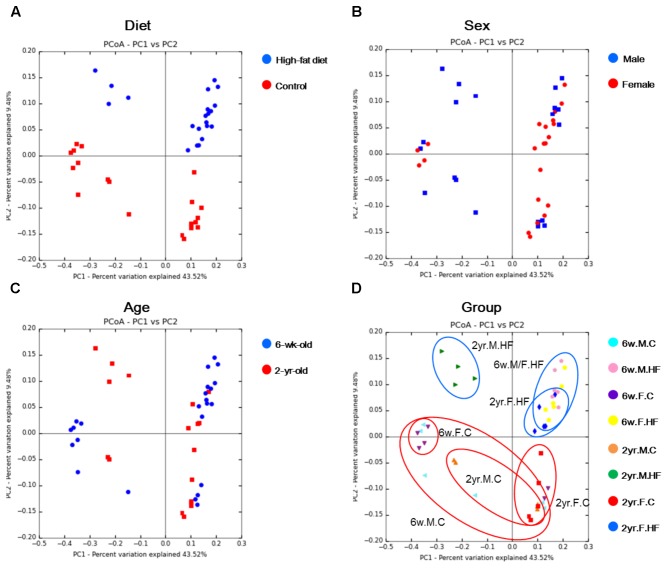
PCoA plots of two ages per diet **(A)**, sex **(B)**, age **(C)**, and experimental groups **(D)**. Metagenome sequencing was performed with 16S rRNA from rat fecal samples. UniFrac-based PCoA was performed on the metagenome sequencing data. 6w.M.C (*n* = 6); 6w.M.HF (*n* = 6); 6w.F.C (*n* = 6); 6w.F.HF (*n* = 6); 2yr.M.C (*n* = 4); 2yr.M.HF (*n* = 4); 2yr.F.C (*n* = 5); 2yr.F.HF (*n* = 6). 6w, 6-week-old; 2yr, 2-year-old; M, male; F, female; C, control; HF, high-fat diet.

The less calorie intake and insignificant change in body weight in 2-year-old male rats may have resulted from the sex differences of food habits. In a previous study, young and growing (4-week old) F344 male rats tended to regulate their energy intake according to their energy requirements (Togo et al., 2012). We assume that the consistent level of calorie intake in male rats may have been due to the modulation of energy intake by the rats. The female-specific increase of calorie intake on HFD feeding in this study newly suggests sex differences in the modulation of calorie intake, although such a conclusion is limited by the small sample size. This less calorie intake of 2-year-old rats may have resulted in distinct separation of gut microbiota of the HFD-fed rats, as shown in **Figure 2D**. The differences of microbiome appeared in specific species, such as the differential levels of the enrichment of *A. muciniphila* and *Desulfovibrio* spp. in 2-year-old male rats (**Figures 4E,G**). That is, there was insignificant change (*A. muciniphila*) or reduction (*Desulfovibrio* spp.) of *A. muciniphila* and *Desulfovibrio* spp. in 2-year-old male rats, which was different from other age and sex. However, the increase of inflammation and cell proliferation in colon mucosa (**Figures 5E,F**) showed that the colon mucosa of 2-year-old rats was significantly influenced by the HFD.

### Gut Microbial Changes Occurring in Rats of Different Sex and Age Following a High-Fat Diet

#### Clustering Analyses

The number of reads of the metagenome sequencing data was 13,087 ± 402.47 (mean ± SEM) reads. When the gut microbiomes of all samples were analyzed in the PCoA, there was clear separation per diet, but not per sex and age (**Figure 2**). The PCoA plots showed clustering by diet in unweighted UniFrac (**Figure 2A**). Samples were separated into the control and HFD groups by PC1 (percent variation explained: 43.52%) and PC2 (9.48%) (**Figure 2A**). Samples were not clearly separated according to neither sex nor age (**Figures 2B,C**). In HFD-fed 2-year-old rats, the samples were segregated depending on sex, mainly by PC1 (43.52%) in unweighted UniFrac (**Figure 2D**). In addition to PCoA, the UPGMA tree of the samples is shown in **Supplementary Figure S2**. The UPGMA tree shows sample clustering by diet, and samples categorized by age. Samples of the HFD groups of 2-year-old rats were separated by sex as well.

Principal component analysis biplots showed alteration in the major components of microbiome caused by HFD (**Supplementary Figure S3**). The major contributing factors for the sample separation by HFD differed according to sex as well as age. In particular, the effect of *Bacteroidetes* appeared to increase in 2-year-old rats when compared with 6-week-old rats.

Sample segregation by diet in the unweighted UniFrac suggested a strong association between HFD and fecal microbiome. The PCoA plots according to groups suggest that the response of gut microbiota to HFD differed according to sex in old age. However, considering the limited number of rats examined in this study, this trend should be verified in a larger population of rats.

#### Changes in Microbial Diversity by HFD in Young and Old Rats

**FIGURE 3:**
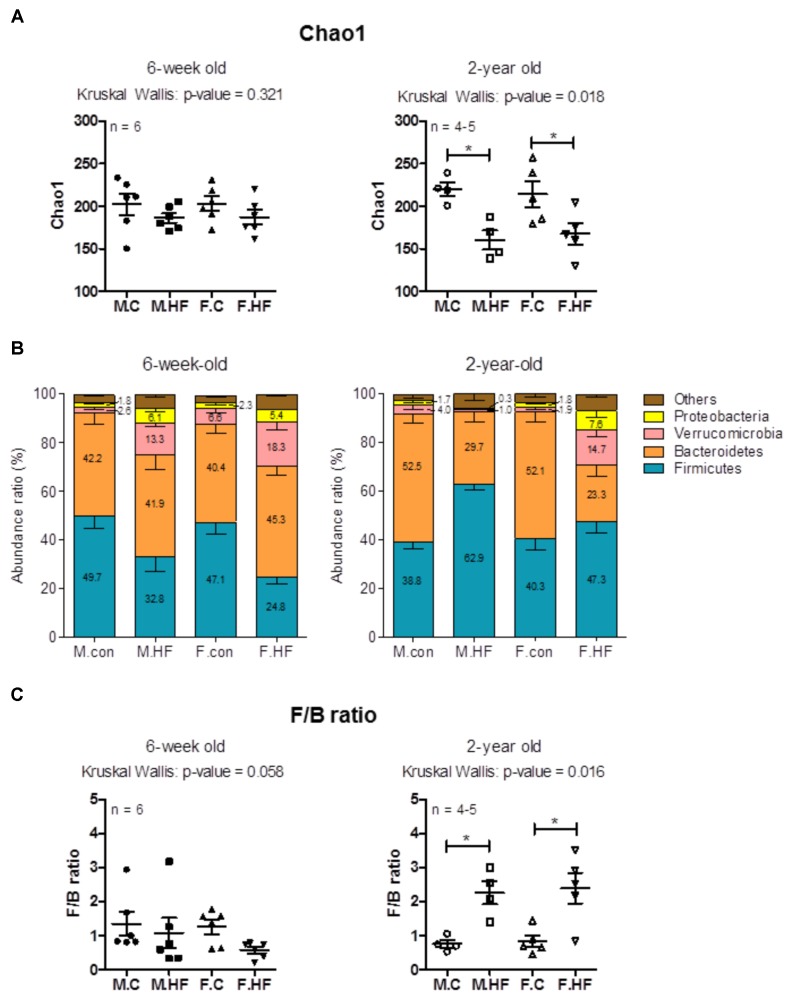
Analysis of species richness and relative abundance ratios of 6-week- and 2-year-old rats. **(A)** Species richness (Chao1 index) of the microbial community determined using pyrosequencing. **(B)** Taxonomic composition at the phylum level. The abundance ratio (%) indicates the percentage of each phylum in total microorganisms. Stacked bars of mean and SEM. **(C)** *Firmicutes*/*Bacteroidetes* ratio calculated by dividing the abundance ratio of *Firmicutes* with that of *Bacteroidetes* in 6-week- and 2-year-old rats. (**A** and **C**) Scatter plot (mean, SEM) (6-week-old rats, *n* = 6; 2-year-old male rats, *n* = 4; 2-year-old female rats, *n* = 5). *p*-Values to Kruskal–Wallis test is designated on the figure; M, male; F, female; C, control; HF, high-fat diet; F/B ratio, *Firmicutes*/*Bacteroidetes* ratio.

High-fat diet feeding negatively influenced the alpha diversity of gut microbiome in old rats regardless of sex. The Chao1 index significantly decreased in 2-year-old rats fed a HFD without any significant changes in the corresponding young rats (*p* = 0.321 in 6w and *p* = 0.018 in 2yr to Kruskal–Wallis test; 6w male: *p* = 0.150; 6w female: *p* = 0.262; 2yr male: *p* = 0.021; 2yr female: *p* = 0.028) (**Figure 3A**). The rarefaction curve of Chao1, which indicates species richness is shown in **Supplementary Figure S4**.

Substantial alpha diversity is a feature of gut microbiome in healthy individuals (Le Chatelier et al., 2013), and low gut microbial diversity is related to obesity (Turnbaugh et al., 2009) and inflammatory bowel disease (Ott and Schreiber, 2006). Thus, the reduction of species richness by HFD in the older age group may suggest an increased risk of metabolic or inflammatory disease. In humans, the microbiota of elderly comprises a less diverse composition than young healthy microbiota (Zwielehner et al., 2009); and diminished microbial diversity correlates with more frailty (Claesson et al., 2012). In addition, subjects with long longevity tend to have high diversity (Kong et al., 2016). In the present study, in rats, there was no significant difference between 6-week old and 2-year old rats (Mann–Whitney with Holm–Bonferroni correction: *p* = 0.394). On the one hand, HFD seemed to induce the decrease of alpha diversity of gut microbiota, especially in old rats; however, the data were not statistically significant due to the small number of rats (Mann–Whitney with Holm–Bonferroni correction: male, *p* = 0.021; female, *p* = 0.028). The reducing pattern of diversity in HFD-fed rats may be relevant to the results in Claesson et al. (2012), which showed highest diversity in “low fat/high fiber” and least diversity in “moderate fat/low fiber” and “high fat/low fiber”.

#### Taxonomic Compositions at Phylum Level

The changes in taxonomic composition by diet were compared between 6-week-old and 2-year-old rats. Phylum level analysis revealed a significant decrease of *Firmicutes* in 6-week-old females by HFD and a marginal decrease in 6-week-old males; in contrast, 2-year-old male rats showed a significant increase in *Firmicutes* by HFD without altering the levels in 2-year-old females (6w male: *p* = 0.055; 6w female: *p* = 0.006; 2yr male: *p* = 0.021; 2yr female: *p* = 0.347) (**Figure 3B**). *Bacteroidetes* levels were diminished by HFD in both male and female 2-year-old rats, but not in 6-week-old males and 6-week-old females (2yr male: *p* = 0.021; 2yr female: *p* = 0.016; 6w male: *p* = 1.000; 6w female: *p* = 0.337). *Proteobacteria* were significantly decreased by HFD in 2-year-old males and significantly increased in 2-year-old female rats, while they showed a marginal increase in 6-week-old rats regardless of sex (2yr male: *p* = 0.043; 2yr female: *p* = 0.016; 6w male: *p* = 0.055; 6w female: *p* = 0.055). Further phylum level analysis showed that the levels of *Verrucomicrobia* spp. were increased by HFD in 6-week-old males and females, and 2-year-old female rats but not in 2-year-old male rats (6w male: *p* = 0.004; 6w female: *p* = 0.016; 2yr female: *p* = 0.009; 2yr male: *p* = 0.248). Other gut microbiota comprising *Actinobacteria*, *Deferribacteres*, *Lentisphaerae*, *Tenericutes*, and unclassified phyla were not altered regardless of age or sex (**Figure 3B**).

Consequently, the HFD significantly increased *Firmicutes*/*Bacteroidetes* ratio particularly in 2-year-old rats not in 6-week-old rats (*p* = 0.016 in 2yr and *p* = 0.058 in 6w to Kruskal–Wallis test; 2yr male: *p* = 0.021; 2yr female: *p* = 0.028; 6w male: *p* = 0.200; 6w female: *p* = 0.078) (**Figure 3C**). In the present study, there were no significant age-related differences in chow-fed rats (6w.M.C vs. 2yr.M.C: *p* = 0.088; 6w.F.C vs. 2yr.F.C: *p* = 0.273, the comparison not shown in the graph).

The HFD-induced decrease of the abundance ratios of *Firmicutes* in 6-week-old rats can be explained with regard to the alterations in the family level. The majorly decreased family in *Firmicutes* was *Ruminococcaceae* (6-week-old male: 18.23 ± 2.30% in control to 4.72 ± 0.84% in HFD, *p* = 0.004; 6-week-old female: 19.39 ± 3.33% in control to 4.08 ± 0.72% in HFD, *p* = 0.004). This alteration is consistent with a previous report (Daniel et al., 2014), which showed the effect of HFD on the gut microbiota composition and the gut bacterial metaproteome in male C57 mice (*n* = 6 per group). This result makes sense regarding the fact that major members of *Ruminococcaceae*, such as *Ruminococci*, utilize plant polysaccharide, regarding that there had been a reduction in amount of polysaccharide in the diet (70%kcal in control to 20%kcal in HFD) in the present study.

An increase in *Firmicutes*/*Bacteroidetes* ratio is a major feature associated with obesity (Mathur and Barlow, 2015). Human aging alters the *Firmicutes*/*Bacteroidetes* ratio: it increases until adulthood and decreases in the elderly, as determined via quantitative PCR (Mariat et al., 2009). Increase of *Firmicutes*/*Bacteroidetes* ratio by HFD in aged rats may be linked to the changes of metabolic state of HFD-fed aged rats; however, since this study has not investigated on the metabolic features, this should be demonstrated through further studies.

#### Taxonomic Composition: Class/Genus/Species

**FIGURE 4:**
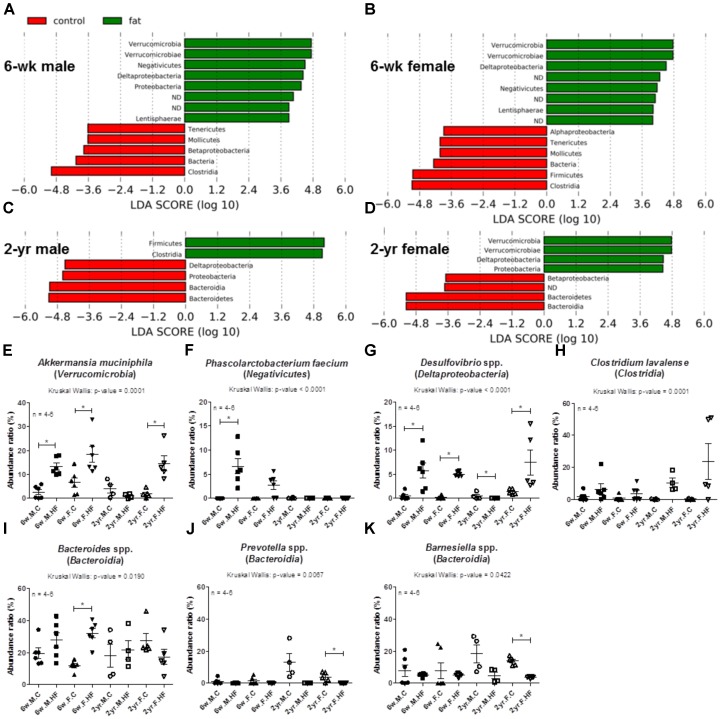
Alterations in the abundance ratio of classes and major species following exposure to high-fat diet. LDA effect size (LEfSe) analyses compared the alterations in microbiome according to the diet at the class level. Negative LDA scores (red) are enriched in controls and positive LDA scores (green) are enriched in high-fat diet groups of 6-week-old male **(A)**, 6-week-old female **(B)**, 2-year-old male **(C)**, and 2-year-old female **(D)**. Abundance ratios (%) of *Akkermansia muciniphila* **(E)**, *Phascolarctobacterium faecium* **(F)**, *Desulfovibrio* spp. **(G)**, *Clostridium lavalense* **(H)**, *Bacteroides* spp. **(I)**, *Prevotella* spp. **(J)**, and *Barnesiella* spp. **(K)** were analyzed according to diet and age. Scatter plot (mean, SEM) (6-week-old rats, *n* = 6; 2-year-old male rats, *n* = 4; 2-year-old female rats, *n* = 5); *p*-values to Kruskal–Wallis test is designated on the figure; ^∗^*p* < 0.05 according to Mann–Whitney *U*-test with Holm–Bonferroni correction; 6w, 6-week-old; 2yr, 2-year-old; M, male; F, female; C, control; HF, high-fat diet.

As analyzed with LEfSe, the following classes were altered by HFD with sufficient abundance ratios: *Verrucomicrobia*, *Negativicutes*, *Deltaproteobacteria*, *Clostridia*, and *Bacteroidia* (**Figure 4**). *Bacteroidia* were consistently decreased by HFD in 2-year-old rats regardless of sex (**Figures 4C,D**). The major genera or species belonging to the phylum or classes exhibiting significant changes in the LEfSe data were further analyzed (**Figures 4E–K**).

The main species belonging to *Verrucomicrobia* was *A. muciniphila*. It was enriched by HFD in 6-week-old males and females, as well as in 2-year-old females, but not in 2-year-old male rats (*p* < 0.001 to Kruskal–Wallis test; 6w male: *p* = 0.004; 6w female: *p* = 0.016; 2yr female: *p* = 0.009; 2yr male: *p* = 0.248) (**Figure 4E**).

Next, the alteration by HFD in the abundance ratios of *Phascolarctobacterium faecium*, belonging to *Negativicutes*, differed according to age. The growth of this species was facilitated by dietary changes, particularly in young rats. The levels of *P. faecium*, were boosted by HFD in 6-week-old rats whereas there was no significant change in 2-year-old rats (*p* < 0.001 to Kruskal–Wallis test; 6w male: *p* = 0.002; 6w female: *p* = 0.022; 2yr male: *p* = 0.317; 2yr female: *p* = 1.000) (**Figure 4F**).

A few potentially pathogenic bacteria were altered by HFD: *Desulfovibrio* spp. and *C. lavalense*. Two *Desulfovibrio* species found in our data, *D. vulgaris* and *D. desulfuricans* are Gram-negative sulfate-reducing bacteria (SRB). The levels of *Desulfovibrio*, comprising *D. vulgaris* and *D. desulfuricans*, were increased by HFD in 6-week-old males and 6-week-old females, as well as in 2-year-old female rats, while they were decreased in 2-year-old male rats (*p* < 0.001 to Kruskal–Wallis test; 6w male: *p* = 0.006; 6w female: *p* = 0.003; 2yr male: *p* = 0.047; 2yr female: *p* = 0.009) (**Figure 4G**). *Clostridia* levels varied at different ages. It comprises 108 species, including *C. lavalense* which was the most abundant *Clostridium* species. *C. lavalense* showed marginal increases by HFD particularly in 2-year-old rats (6w male: *p* = 0.150; 6w female: *p* = 0.522; 2yr male: *p* = 0.021; 2yr female: *p* = 0.076) (**Figure 4H**).

The abundance ratio of *Bacteroidia* in our data was substantiated by alterations in the three dominant genera, *Bacteroides*, *Prevotella*, and *Barnesiella*. The most abundant genus, *Bacteroides*, was significantly increased by HFD in 6-week-old female rats, while there was no significant change in other groups (*p* = 0.019 to Kruskal–Wallis test; 6w male: *p* = 0.200; 6w female: *p* = 0.004; 2yr male: *p* = 0.564; 2yr female: *p* = 0.076) (**Figure 4I**). By contrast, the decrease of *Prevotella* spp. by HFD occurred only in old rats (*p* = 0.007 to Kruskal–Wallis test; 6w male: *p* = 0.618; 6w female: *p* = 0.521; 2yr male: *p* = 0.021; 2yr female: *p* = 0.009) (**Figure 4J**). Next, *Barnesiella* species were significantly decreased by HFD in older females, but only marginally in older males, without varying in young rats (*p* = 0.042 to Kruskal–Wallis test; 6w male: *p* = 1.000; 6w female: *p* = 0.336; 2yr male: *p* = 0.083; 2yr female: *p* = 0.009) (**Figure 4K**).

*Akkermansia muciniphila* are mucin-utilizing bacteria that colonize in the mucin layer (Derrien et al., 2004). Many studies have demonstrated the beneficial effects of *A. muciniphila* on obesity and metabolic profiles. *A. muciniphila* are known to adhere to enterocytes and enhance the integrity of the epithelial cell layer (Reunanen et al., 2015). This species has also been shown to be reduced in response to HFD feeding in mice (Schneeberger et al., 2015). However, in a previous study, the caecal abundance of *Akkermansia* was higher in rats fed with HFD compared with in low-fat diet-fed rats, suggesting the need for caution in proclaiming the probiotic effect of *A. muciniphila* (Zhong et al., 2015). Mucus degradation by *A. muciniphila* may lead to compensatory hyperproliferation and increase the risk of colon cancer, along with the effects of sulfide-producing bacteria (Ijssennagger et al., 2015). On the other hand, *A. muciniphila* has all genes encoding the enzymes involved in the citric acid cycle (TCA cycle), an important pathway for the metabolism of fatty acids, while many species seem to lack genes for the full cycle according to Kyoto Encyclopedia of Genes and Genomes (KEGG) pathways (Kanehisa and Goto, 2000). We considered the fatty acid metabolism of *A. muciniphila* as a contributing factor for the induction of the growth of *A. muciniphila* seen in a few of the HFD groups in our data.

*Phascolarctobacteria* in the human gastrointestinal tract synthesize a substantial amount of SCFA (Wu et al., 2017). In humans, *P. faecium*-like bacteria were maintained sufficiently in young individuals and decreased in the elderly population aged above 60 years (Wu et al., 2017). *P. faecium* was also associated with cruciferous diets in a controlled feeding study in humans (Li et al., 2009). *P. faecium*, which increased in response to HFD in young rats, might have had beneficial effects on the colons of young rats by producing SCFA, which are the energy source of colonic epithelial cells (Dot et al., 1993). However, further studies are required to elucidate this association.

Sulfate-reducing bacteria produce hydrogen sulfide (H_2_S), which has been shown to have cytotoxic and genotoxic effects in cell culture studies (Deplancke and Gaskins, 2003; Attene-Ramos et al., 2006). Sulfidogenic bacteria are a risk factor for colorectal cancer in African Americans, in whom the colorectal cancer incidence is higher than in non-Hispanic whites (Yazici et al., 2017). These results support a possible association between *Desulfovibrio* and colorectal cancer risk.

Currently, there are two reported *C. lavalense* strains, CCRI-9842T and CCRI-9929. CCRI-9842T, which reduces nitrate, was the most accurate matching strain in the present blast results (Domingo et al., 2009). Nitrate is reduced to nitrite by enzymes produced by gut microbiota in order to form *N*-nitroso compounds (NOCs). NOCs are known carcinogens that form DNA adducts inducing mutations. The influence of nitrate-reducing bacteria on gastric cancer has been suggested (Ferreira et al., 2018); yet little is known of the association with colon cancer. Our results suggest the possibility of an association between these potentially pathogenic bacteria and colon carcinogenesis.

The old rats were distinguished by a decline in class *Bacteroidia*. *Bacteroidia* is a class of *Bacteroidetes* and comprises genera that are associated with dietary types (Wu et al., 2011). *Bacteroides* is known to increase in obese and overweight individuals compared with lean controls (Schwiertz et al., 2010). *Bacteroides* have a broad capacity to utilize various types of dietary polysaccharides (Xu et al., 2007). High-carbohydrate diets enrich some saccharolytic bacteria including Bacteroides in people “at risk” of a metabolic syndrome, regardless of glycemic index (Fava et al., 2013). In our study, the abundance of *Bacteroides* elevated with HFD in young females; this result is not consistent with previous reports. Since a significant increase of *Bacteroides* was induced by HFD only in young female rats, this study suggests the possibility that the effect of HFD on *Bacteroides* may vary according to age and sex. Sex-specific effects of carbon source in diet have been demonstrated in fish (Bolnick et al., 2014).

The decrease of *Prevotella* spp. by HFD in old rats is understandable since *Prevotella* are cellulolytic bacteria. The microbiota enriched with *Prevotella* has been related with carbohydrate-based diets (Wu et al., 2011). In this study, the carbohydrate level was reduced in the HFD. In addition to the effects of high fat proportion in diets, the lower percentage of carbohydrates can exert an impact on the gut microbiome (Fava et al., 2013). We presume that the reduction of carbohydrates in the diets has influenced on the decrease of *Prevotella* in the HFD groups, specifically in old rats. On the other hand, the *Bacteroides*/*Prevotella* ratios significantly decreased in the highly frail elderly population compared to in the low-frail elderly individuals (van Tongeren et al., 2005). The association of the genus with aging and frailty is yet to be elucidated.

This result is consistent with previous literatures. One study showed a decrease of *Barnesiella*, a SCFA-producing bacteria, in the colon of HFD-fed mice, which may be linked to the gut inflammatory phenotype in azoxymethane-induced colon cancer (Zeng et al., 2018). Likewise, the present study shows the age-specific reduction of potentially beneficial *Barnesiella* spp. by HFD in older rats.

### Host Response in Colon Occurring in Rats of Different Sex and Age Following a High-Fat Diet

#### Histological Analysis of Fat Deposition

We analyzed the histological features of host colons including fat accumulation, inflammation, and cell proliferation, and compared them according to age, sex, and diet.

**FIGURE 5:**
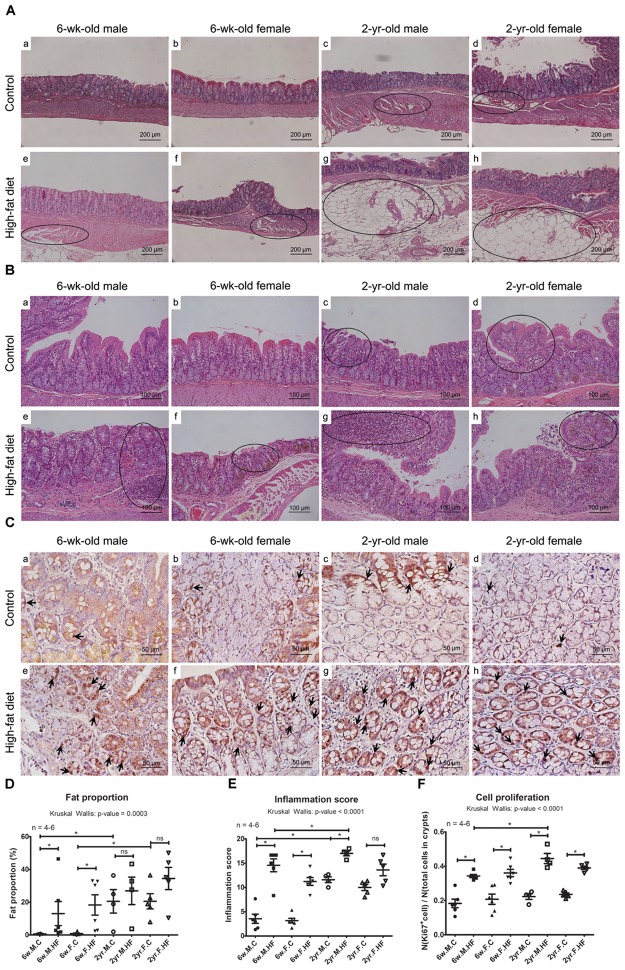
Histological analysis of colonic tissues. Fat deposition **(A)** and inflammation **(B)** in the photomicroscopy of H&E stain of ascending colons (**A**: ×200 magnifications; **B**: ×100 magnifications). Dotted circles represent regions with fat deposition or inflammation. **(C)** Immunohistochemical images of Ki67 (×400 magnifications). Arrows indicate representative Ki67-immunoreactive cells. **(D)** Fat proportion (%) in total smooth muscle area. **(E)** Inflammation scores of colonic epithelial damage and inflammatory cell infiltration in mucosa, submucosa, and muscle layers. **(F)** The Ki67 proliferation index calculated by dividing the number of Ki67-stained cells with the number of total cells within all crypts. Scatter plot (mean, SEM) (6-week-old rats, *n* = 6; 2-year-old male rats, *n* = 4; 2-year-old female rats, *n* = 5). *p*-Values to Kruskal–Wallis test is designated on the figure; ^∗^*p <* 0.05 according to Mann–Whitney *U*-test with Holm–Bonferroni correction. M, male; F, female; C, control; HF, high-fat diet; Control groups of 6-week-old male **(a)**, 6-week-old female **(b)**, 2-year-old male **(c)**, 2-year-old female **(d)**, and high-fat diet groups of 6-week-old male **(e)**, 6-week-old female **(f)**, 2-year-old male **(g)**, and 2-year-old female **(h)**.

In the case of 6-week-old HFD-fed rats, the average fat proportion significantly increased with HFD (*p* < 0.001 to Kruskal–Wallis test; 6w male: *p* = 0.004; 6w female: *p* = 0.006; 2yr male: *p* = 0.386; 2yr female: *p* = 0.175) (**Figures 5A-e,-f,D**). In addition, the fat proportion was higher in the 2-year-old control rats than in the 6-week-old control rats (male: *p* = 0.011; female: *p* = 0.006).

The high fat proportion in the 2-year-old control groups resulted in the insignificant difference between control and HFD groups in old rats. In our previous study, we identified the fat deposition in the muscle layer of ascending colons as a feature of aged colons, which resulted in age-related colonic dysmotility (Jo et al., 2014; Lee et al., 2017). This study found that HFD induced fat deposition in tunica muscularis in a few young rats, although there were individual variations among rats. These results suggest that the increased fat proportion in the muscle layers may contribute to HFD-associated colonic dysmotility as well as age-related colonic dysmotility. The delayed colonic transits make luminal contents remain longer in colons, so it may induce microbial alterations in HFD-fed or aged rats. Conversely, intestinal dysbiosis contributes to the HFD-associated delay of gastrointestinal transit via neuronal loss (Anitha et al., 2016), and to chronic constipation via the upregulation of serotonin transporter expression (Cao et al., 2017).

#### Histological Inflammation Score

This study compared the effects of HFD in young and old rats. The histological inflammation scores significantly increased with HFD in young rats and male aged rats, but not in female aged rats (*p* < 0.01 to Kruskal–Wallis test; 6w male: *p* = 0.004; 6w female: *p* = 0.004; 2yr male: *p* = 0.021; 2yr female: *p* = 0.059) (**Figures 5B-a–h,E**). In the control groups, the inflammation scores were increased with age (male: *p* = 0.011; female: *p* = 0.006) (**Figures 5B-a–d**).

#### Cell Proliferation in Colonic Mucosa

Immunohistochemical staining for Ki67 revealed that Ki67-positive cells were increased by exposure to HFD in all age and sex (*p* < 0.001 to Kruskal–Wallis test; 6w male: *p* = 0.004; 6w female: *p* = 0.004; 2yr male: *p* = 0.021; 2yr female: *p* = 0.009) (**Figures 5C,F**). The percentage of Ki67-stained cells was significantly higher in the 2-year-old rats compared to in the 6-week-old rats exposed to HFD, significantly in males and marginally in females (male: *p* = 0.011; female: *p* = 0.021) (**Figures 5C-e–h**).

Ki67 is a widely used proliferation marker, which is closely associated with colorectal cancer risk (Lipkin, 1988). Thus, we determined the proportion of Ki67-positive cells among the total cells constituting the crypts of colonic mucosa by IHC staining, and found an increase in the number of Ki67-postive cells by HFD. In addition, the percentage of Ki67-positive cells was higher in old rats than in young rats in the HFD-fed groups. These results demonstrate that HFD promotes cell proliferation in colonic mucosa and does so more significantly in old age. This is consistent with the increase in the bile acid homeostasis, cell proliferation, and tumorigenesis in the colons of mice exposed to a Western diet (a diet in which plant-based fat is substituted with anhydrous milk fat) (Dermadi et al., 2017). In addition, we suggest a greater impact of HFD on the dysregulation of cell proliferation in old age than in young age.

### Association Between the Gut Microbiota and Colon Mucosa

#### Correlation Between Characteristics of Gut Microbiota and Ki67 Proliferation Index

**FIGURE 6:**
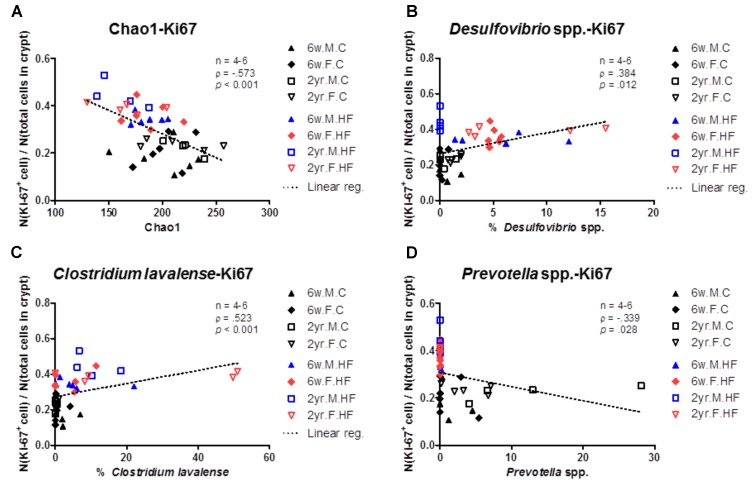
Correlation between gut microbiome and cell proliferation index. Species richness (Chao1) **(A)**, abundance ratios (%) of *Desulfovibrio* spp. **(B)**, *Clostridium lavalense* **(C)**, and *Prevotella* spp. **(D)** were significantly correlated with cell proliferation index of colonic mucosa (N(Ki67 + cell)/*N*(total cells in crypts). *p* < 0.05 to Spearman’s correlation **(A–D)**. Dotted line indicates the regression line. The correlations were analyzed with Spearman’s rank correlation coefficient method (also known as Spearman’s rho) and linear regression.

The species richness (Chao1 index) was significantly correlated with the cell proliferation index of colonic mucosa regardless of age or sex, showing a negative correlation (Spearman’s rho = -0.573, *p <* 0.0001) (**Figure 6A**). The abundance ratios of *Desulfovibrio* spp., *C. lavalense*, and *Prevotella* spp. were also significantly correlated with the proliferation index (*Desulfovibrio* spp., Spearman’s rho = 0.417, *p* = 0.006; *C. lavalense*, Spearman’s rho = 0.523, *p <* 0.0001; *Prevotella* spp., Spearman’s rho = -0.339, *p* = 0.127) (**Figures 6B–D**).

These results reveal the association between dysbiosis markers and Ki67 cell proliferation index in colon mucosa. As discussed above, *Desulfovibrio* spp. are sulfidogenic bacteria and *C. lavalense* are nitrate-reducing bacteria. Since these bacteria produce the genotoxic or carcinogenic materials, we suggest that the increase of *Desulfovibrio* spp. and *C. lavalense* in HFD-fed old rats may contribute to the up-regulation of cell proliferation in colon mucosa.

#### Inflammation and Proliferation Markers in Colonic Mucosa

**FIGURE 7:**
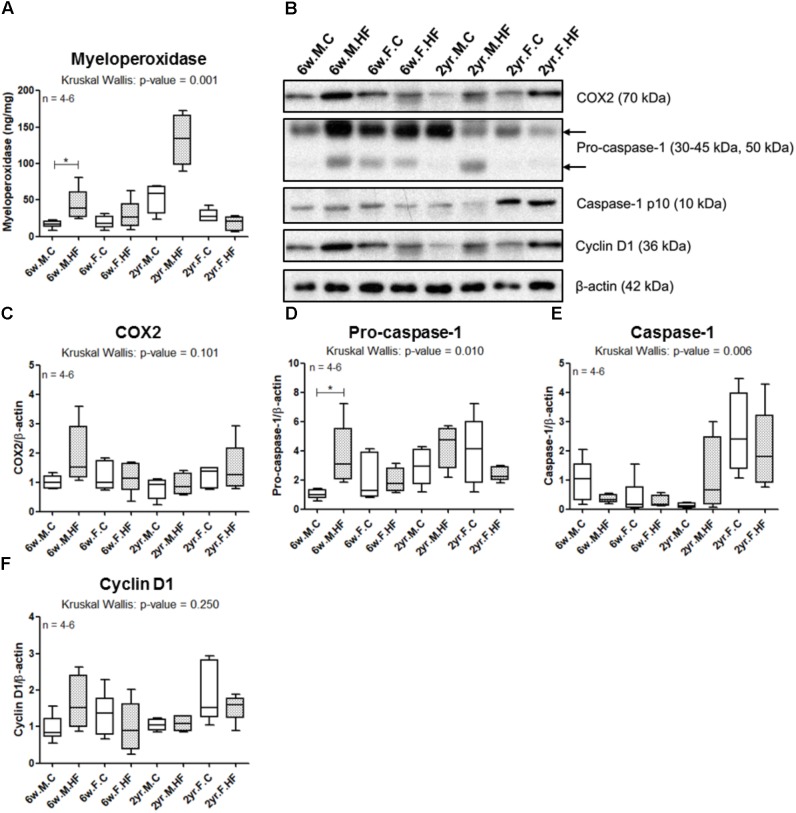
Inflammation and proliferation markers in colonic mucosa. Myeloperoxidase concentration **(A)** was assessed by ELISA. **(B)** Western blot of COX2, pro-caspase-1, caspase-1 p10, and cyclin D1 was performed with colonic mucosa samples. Band intensities of COX2 **(C)**, pro-caspase-1 **(D)**, caspase-1 p10 **(E)**, and cyclin D1 **(F)** were quantified by densitometry, adjusted to that of β-actin, an endogenous control, and normalized to the mean value of 6w.M.C group. Box and whiskers (Turkey): the central line on each box, median; the edges of boxes, 25th and 75th percentiles; whiskers, maximum and minimum values of the data. *p*-Values to Kruskal–Wallis test are designated on the figure; ^∗^*p* < 0.05 according to Mann–Whitney *U*-test with Holm–Bonferroni correction. 6w, 6-week-old; 2yr, 2-year-old; M, male; F, female; C, control; HF, high-fat diet.

We evaluated the effects of HFD on colonic mucosal inflammation, based on the expression of markers of inflammation. A sex difference was observed in MPO concentration, as the MPO concentration in colonic mucosa was increased by HFD particularly in young male rats; conversely, in female rats, there was no significant change in response to HFD (*p* = 0.001 to Kruskal–Wallis test; 6w male: *p* = 0.006; 6w female: *p* = 0.273; 2yr male: *p* = 0.021; 2yr female: *p* = 0.175) (**Figure 7A**). The protein expression levels of COX2 showed no significant difference (*p* = 0.101 to Kruskal–Wallis test) (**Figures 7B,C**). Similar to the MPO, the level of pro-caspase-1 showed a significant increase by HFD only in 6-week-old male rats (*p* = 0.010 to Kruskal–Wallis test; 6w male: *p* = 0.006; 6w female: *p* = 0.670; 2yr male: *p* = 0.149; 2yr female: *p* = 0.251) (**Figures 7B,D**). However, although the level of caspase-1 p10 differed among the eight groups (*p* = 0.006 to Kruskal–Wallis test), it showed no significant difference in the multiple comparison (6w male: *p* = 0.197; 6w female: *p* = 0.670; 2yr male: *p* = 0.149; 2yr female: *p* = 0.251) (**Figures 7B,E**). The protein expression levels of cyclin D1 showed no significant difference (*p* = 0.250 to Kruskal–Wallis test) (**Figures 7B,F**). In multiple comparison analysis by Mann–Whitney test with Holm–Bonferroni correction, although the *p*-values were considered insignificant due to the small number of subjects, the expression levels of COX2 and cyclin D1 were most altered by HFD in 6-week-old male rats (COX2: *p* = 0.045; cyclin D1: *p* = 0.028) (**Figures 7B,F**).

The HFD-induced increase of MPO in young males was consistent with conventional findings that revealed the direct effect of HFD on host inflammation in young males. HFD is known to induce the formation of precursors of colon cancer including proliferating cell nuclear antigen (PCNA), cyclin D1, NF-κB proteins, and β-catenin, in 4-week-old male Wistar rats (Zhu et al., 2014). In addition, HFD-induced obesity promotes the formation of colitis-associated tumors in the colons of 4-week-old male A/J mice (Park et al., 2012). HFD-induced systemic inflammation is related to the increased endotoxin levels via altered composition of gut microbiota, according to a study of 4-week-old male mice (Kim et al., 2012). However, these studies were restricted to responses in young males.

Cyclin D1 is a regulator of G_1_-to-S transition, and is overexpressed in diverse human cancers (Hunter and Pines, 1994). In our data, the expression of Ki67 in IHC and the expression of cyclin D1 in Western blots showed different patterns among the experimental groups. While Ki67 was consistently increased by HFD across all age and sex groups, cyclin D1 was increased by diet only in young males. We suggest two major reasons for the varying expression of the two markers. First, the two different cell cycle markers indicate different ranges of cell cycles: while Ki67 is a pan-cell cycle marker, cyclin D1 is a marker of G1-phase. Second, the differences in techniques used: while the expression of Ki67 was detected by counting the immunoreactive cells only within crypts, the expression of cyclin D1 was measured using the whole proteins of colonic mucosa. The increase of cyclin D1 protein expression by HFD in colonic mucosa of young males is consistent with a report investigating the cyclin D1 levels in colonic mucosa of 4-week-old male mice following exposure to HFD (45% kcal as fat) (Park et al., 2012).

### Links Between Aging, Sex, Diet, Gut Microbiota, and Colon Cancer

The fat accumulation and inflammation in colons are related to the aging properties of colon. The histological fat proportion in muscle layers of colons is linked to colon dysmotility, a feature of aging colon (Jo et al., 2014; Lee et al., 2017). The colon dysmotility is presumed to lead to alterations of gut microbiota due to a longer stay of intestinal contents in the gut. In addition, inflammation and cell proliferation of colon mucosa are related to the susceptibility for colon cancer. That is, inflammation promotes colon cancer development (Terzić et al., 2010); and increased mucosal proliferation has been associated with a higher colorectal cancer risk (Lipkin, 1988). This study suggest that the colons of aged males, with higher fat proportion in muscle layer and inflammation in colon mucosa, may be more vulnerable to dietary effect, to reach the highest cell proliferation in colon mucosa.

The pro-inflammatory effects of HFD have been observed in young rats and male aged rats. Previous results have suggested that HFD promoted low-grade intestinal inflammation in mice (Ding et al., 2010; Kim et al., 2012). In addition, it has been suggested that dietary fat aggravates colitis induced by dextran sulfate sodium, by elevating pro-inflammatory cytokines (Okada et al., 2013). The inflammatory features of aged colons in control and in HFD groups were attributed to the effects of inflamm-aging and immunosenescence. Interaction between gut microbiota and the host is an important factor in preserving host health against inflammatory diseases and colon cancer (Sansonetti, 2010). It has been known that dysbiosis results in immunological dysregulation and inflammation in old age (Guigoz et al., 2008; Round and Mazmanian, 2009). Specifically, the present study revealed significant correlations between the gut dysbiosis markers and the cell proliferation of colon mucosa indicated by the histological expression of Ki67. Therefore, in addition to previous findings, we suggest specific factors linking the composition of gut microbiota with the risk of colon carcinogenesis.

Here we discuss the sex differences in inflammatory responses including immune reaction to HFD. Histological observations in this study showed that inflammation and cell proliferation of colon mucosa were similarly induced by HFD in both sexes. However, the levels of MPO, which are the molecular factors related to neutrophils, were lower in females. The MPO activity is linked with the severity of dextran sulfate sodium-induced colitis (Vowinkel et al., 2004), and its expression is an indicating factor of colon cancer risk (Roncucci et al., 2008). The increase of caspase-1 in old females may indicate the activation of NLR family pyrin domain containing 3 (NLRP3) inflammasome resulting in pyroptosis (Miao et al., 2011), which triggers anti-cancer immune responses (Kepp et al., 2010). Therefore, we suggest that the underlying mechanisms of inflammation and immune responses may vary between males and females. Induction of the neutrophil activation may be higher in males, while other pathways may mediate inflammation in females.

The microbiome research has been increasingly recognized to be important, as seen in the goals of the new EU Health and Food Work Programs for 2018–2020 to expand the knowledge on the microbiome, nutrition, host health, and disease (Hadrich, 2018). Understanding the influence of microbiota composition and microbial activities is important since they interact with host metabolism and contribute to development of disease such as inflammatory bowel diseases, obesity, and metabolic syndrome (Ottman et al., 2012). Dietary alteration changes functional gene profiles of microbiota, including bacterial protein export, secretion system, and metabolism of lipoic acid (Wu et al., 2011). For instance, a recent study showed that the genus *Prevotella* was negatively correlated with fat intake in fecal microbiota of children from rural and urban areas in Philippines, which was consistent with our results (Nakayama et al., 2017). In addition to the previous knowledge, the present study demonstrates a negative correlation between the *Prevotella* and the colon cancer risk as well as the positive correlations between the increase of some pathogenic bacteria and the risk of colon cancer. These results are relevant to a previous report that demonstrated the effect of dietary heme on gut dysbiosis and the incidence and progression of chemically induced colitis and colorectal cancer (Constante et al., 2017); since Western diet is featured with a high intake of red meat which is rich in both fat and heme. While the present study has focused on the responses in the colonic mucosa, commensal bacteria contribute to systemic immune responses as well as the responses in gut (Kosiewicz et al., 2011; Pindjakova et al., 2017). We assume that these approaches studying the correlation between diet-induced dysbiosis and the human diseases may prompt the implication of the microbiome research on human health.

## Conclusion

The present study showed age- and sex-related differences in the response of gut microbiota and host colons following exposure to HFD in F344 rats, and the correlation between microbial changes and host response. To briefly summarize the major results, the decrease in species richness and the increase in *Firmicutes*/*Bacteroidetes* ratio by HFD occurred only in the gut microbiota of aged rats, not in that of young rats. This study confirmed that aging induces fat accumulation in colonic muscle layers, and inflammation in colon mucosa. The Ki67 cell proliferation index in colonic mucosa was increased with HFD, and the rate was higher in aged male rats than in young male rats. The species richness (Chao1) and the abundance ratios of potentially pathogenic species (*Desulfovibrio* spp. and *C. lavalense*) in the gut microbiome were significantly correlated with Ki67 cell proliferation index in colon mucosa. Sex differences in the alteration by HFD in the gut microbiome were observed in the microbiome of aged rats. For instance, *A. muciniphila* and *Desulfovibrio* spp. increased in response to HFD in young rats and female aged rats, but not in male aged rats. Among the inflammatory and proliferation markers (MPO, COX2, caspase-1, and cyclin D1), the MPO concentrations showed varying HFD response according to age and sex; the increase in the expression of MPO was more prominent in males than that in females.

The results cannot explain the cause-and-effect relationship between the microbial changes and host responses during HFD feeding, and this is a limitation of the study. However, the associations in this study suggest that some dysbiosis factors are negatively correlated with colonic health, and may vary according to age and sex. In addition, this study has limitation that the rats were influenced by coprophilic behaviors, since two rats in the same group were cohoused per cage. However, despite the pairing of the microbiome of two rats from the same cage, we could find clustering of microbiome according to diet in PCoA plots. To verify the results of this study, further studies with a larger number of rats housed in independent cages are needed.

In conclusion, the results suggest a link between HFD-induced gut dysbiosis (particularly the low species richness and high abundance ratios of *Desulfovibrio* spp. and *C. lavalense*) and cell proliferation of colon mucosa (indicated by Ki67 IHC). In addition, sex differences influence on the response of gut microbiome to HFD particularly in old age, and this sex difference in the gut microbiota might be linked to the sex differences of inflammation in the colon mucosa.

## Data Availability

The datasets generated for this study can be found in the NCBI Sequence Read Archive Database (GEO accession number GSE104184) (https://www.ncbi.nlm.nih.gov/geo/query/acc.cgi?acc=GSE104184).

## Author Contributions

NK designed the research. SL and RN performed the research. SL and HY analyzed the data. SL wrote the paper. NK and DL supervised the manuscript.

## Conflict of Interest Statement

The authors declare that the research was conducted in the absence of any commercial or financial relationships that could be construed as a potential conflict of interest.

## Footnotes

1 http://huttenhower.sph.harvard.edu/galaxy/

2 https://www.ncbi.nlm.nih.gov/geo/query/acc.cgi?acc=GSE104184
