# Supplementary material for: Microbial Changes and Host Response in F344 Rat Colon Depending on Sex and Age Following a High-Fat Diet
Source: Front Microbiol. 2018 Sep 21;9:2236. doi: 10.3389/fmicb.2018.02236 (PMC6160749; doi:10.3389/fmicb.2018.02236)
Supplement: Supplementary file 1 [file Table_1.DOCX]

Supplementary Material

Microbial changes and host response in F344 rat colon depending on sex and age following a high-fat diet

Sun Min Lee, Nayoung Kim*, Hyuk Yoon, Ryoung Hee Nam, Dong Ho Lee

*** Correspondence:** Nayoung Kim: nakim49@snu.ac.kr

# Supplementary Table S1. Diet composition

| Formulas (Product #) | 60 kcal% fat  (D12492) | 10kcal % fat  (D12450J) |
| --- | --- | --- |
|  | kcal% | kcal% |
| Protein | 20 | 20 |
| Carbohydrate | 20 | 70 |
| Fat | 60 | 10 |
| Soybean Oil | 6 | 6 |
| Lard | 54 | 4 |
| Total | 100 | 100 |
