# Supplementary material for: Microbial Changes and Host Response in F344 Rat Colon Depending on Sex and Age Following a High-Fat Diet
Source: Front Microbiol. 2018 Sep 21;9:2236. doi: 10.3389/fmicb.2018.02236 (PMC6160749; doi:10.3389/fmicb.2018.02236)
Supplement: Supplementary file 2 [file Table_2.DOCX]

Supplementary Material

Microbial changes and host response in F344 rat colon depending on sex and age following a high-fat diet

Sun Min Lee, Nayoung Kim*, Hyuk Yoon, Ryoung Hee Nam, Dong Ho Lee

*** Correspondence:** Nayoung Kim: nakim49@snu.ac.kr

# Supplementary Table S2. Detailed quality of reads

| **Result of Merge (by FLASH)** | | | | | | |
| --- | --- | --- | --- | --- | --- | --- |
| **SampleName** | **Total Bases** | **Read Count** | **N (%)** | **GC (%)** | **Q20 (%)** | **Q30 (%)** |
| 6w.M.C1 | 59,996,123 | 134,087 | 0.0005 | 54.05 | 96.68 | 90.1 |
| 6w.M.C2 | 64,965,917 | 145,422 | 0.0007 | 53.23 | 96.76 | 90.32 |
| 6w.M.C3 | 63,667,182 | 142,331 | 0.0006 | 51.62 | 96.7 | 90.17 |
| 6w.M.C4 | 59,811,718 | 133,936 | 0.0005 | 51.88 | 96.62 | 89.95 |
| 6w.M.C5 | 57,710,561 | 128,634 | 0.0005 | 52.48 | 96.49 | 89.73 |
| 6w.M.C6 | 59,047,076 | 132,375 | 0.0003 | 52.05 | 96.01 | 88.52 |
| 6w.M.HF1 | 65,321,918 | 144,138 | 0.0005 | 52.67 | 96.23 | 89.17 |
| 6w.M.HF2 | 53,915,820 | 119,478 | 0.0004 | 52.63 | 96.53 | 89.79 |
| 6w.M.HF3 | 61,811,964 | 136,860 | 0.0007 | 53.08 | 96.51 | 89.85 |
| 6w.M.HF4 | 51,248,164 | 115,068 | 0.0006 | 53.49 | 96.95 | 90.82 |
| 6w.M.HF5 | 60,290,885 | 133,771 | 0.0003 | 53.35 | 96.47 | 89.67 |
| 6w.M.HF6 | 59,525,274 | 132,328 | 0.0003 | 52.47 | 96.54 | 89.84 |
| 6w.F.C1 | 60,794,410 | 135,467 | 0.0004 | 53.72 | 96.65 | 89.99 |
| 6w.F.C2 | 60,449,550 | 134,220 | 0.0004 | 53.42 | 96.27 | 89.18 |
| 6w.F.C3 | 66,963,845 | 150,247 | 0.0005 | 52.38 | 96.79 | 90.36 |
| 6w.F.C4 | 55,417,216 | 124,392 | 0.0005 | 52.46 | 96.73 | 90.19 |
| 6w.F.C5 | 60,034,023 | 134,613 | 0.0006 | 52.03 | 96.85 | 90.49 |
| 6w.F.C6 | 66,030,272 | 147,888 | 0.0004 | 52.4 | 96.87 | 90.61 |
| 6w.F.HF1 | 62,512,098 | 137,831 | 0.0003 | 52.63 | 96.39 | 89.54 |
| 6w.F.HF2 | 63,298,289 | 140,812 | 0.0003 | 53.6 | 96.62 | 89.92 |
| 6w.F.HF3 | 63,243,328 | 141,026 | 0.0005 | 53.22 | 96.75 | 90.26 |
| 6w.F.HF4 | 59,840,344 | 133,742 | 0.0004 | 53.25 | 96.6 | 89.93 |
| 6w.F.HF5 | 67,762,133 | 150,189 | 0.0005 | 52.72 | 96.61 | 89.95 |
| 6w.F.HF6 | 53,785,940 | 119,796 | 0.0004 | 52.72 | 96.77 | 90.3 |
| 2yr.M.C1 | 54,812,393 | 121,619 | 0.0005 | 54.37 | 96.49 | 89.77 |
| 2yr.M.C2 | 54,193,983 | 121,053 | 0.0004 | 54.1 | 96.7 | 90.18 |
| 2yr.M.C3 | 48,840,568 | 108,481 | 0.0001 | 53 | 95.05 | 86.37 |
| 2yr.M.C4 | 56,736,671 | 125,904 | 0.0002 | 52.87 | 96.46 | 89.66 |
| 2yr.M.HF1 | 46,269,150 | 103,926 | 0.0005 | 53.93 | 96.6 | 89.91 |
| 2yr.M.HF2 | 50,625,785 | 113,592 | 0.0004 | 53.76 | 96.74 | 90.26 |
| 2yr.M.HF3 | 44,590,931 | 100,237 | 0.0005 | 53.14 | 96.77 | 90.25 |
| 2yr.M.HF4 | 59,139,675 | 132,991 | 0.0007 | 53.34 | 96.91 | 90.71 |
| 2yr.F.C1 | 47,485,510 | 106,130 | 0.0005 | 53.13 | 96.77 | 90.37 |
| 2yr.F.C2 | 67,665,772 | 150,951 | 0.0004 | 52.72 | 96.43 | 89.51 |
| 2yr.F.C3 | 77,207,320 | 171,820 | 0.0005 | 53.1 | 96.69 | 90.19 |
| 2yr.F.C4 | 76,132,211 | 169,184 | 0.0006 | 52.64 | 96.45 | 89.53 |
| 2yr.F.C5 | 65,052,185 | 144,826 | 0.0004 | 53.68 | 96.49 | 89.74 |
| 2yr.F.HF1 | 67,592,300 | 151,235 | 0.0003 | 53.23 | 96.51 | 89.67 |
| 2yr.F.HF2 | 42,324,125 | 94,934 | 0.0002 | 54.07 | 96.62 | 89.9 |
| 2yr.F.HF3 | 50,106,202 | 112,844 | 0.0004 | 53.9 | 97 | 90.83 |
| 2yr.F.HF4 | 55,843,148 | 125,510 | 0.0003 | 53.5 | 96.97 | 90.81 |
| 2yr.F.HF5 | 61,235,188 | 137,112 | 0.0003 | 54.38 | 96.74 | 90.18 |
