# Supplementary material for: Microbial Changes and Host Response in F344 Rat Colon Depending on Sex and Age Following a High-Fat Diet
Source: Front Microbiol. 2018 Sep 21;9:2236. doi: 10.3389/fmicb.2018.02236 (PMC6160749; doi:10.3389/fmicb.2018.02236)
Supplement: Supplementary file 5 [file Image_1.PDF]

Supplementary Material

Microbial changes and host response in F344 rat colon depending on sex and age following a high-fat diet

Sun Min Lee, Nayoung Kim*, Hyuk Yoon, Ryoung Hee Nam, Dong Ho Lee

*** Correspondence:** Nayoung Kim: nakim49@snu.ac.kr

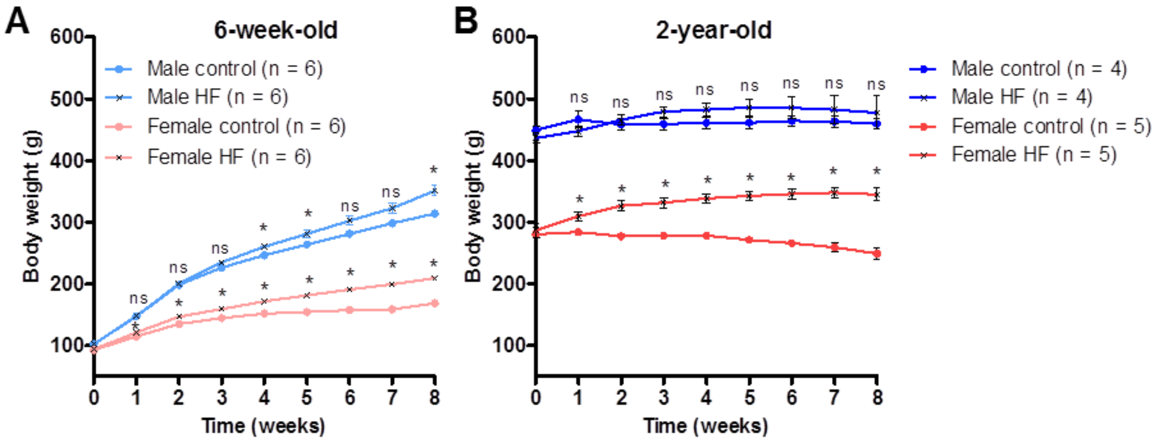

Supplementary Figure S1. Growth curve of rats during the feeding period (8 weeks) (6-week-old rats, n = 6; 2-year-old male rats, n = 4; 2-year-old female rats, n = 5). (A) 6-week-old, (B) 2-year-old. Data are expressed as mean and SEM; $^{*}p < 0.05$ to Mann-Whitney U test, control vs. HF; M, male; F, female; HF, high-fat diet.
