# Supplementary material for: Microbial Changes and Host Response in F344 Rat Colon Depending on Sex and Age Following a High-Fat Diet
Source: Front Microbiol. 2018 Sep 21;9:2236. doi: 10.3389/fmicb.2018.02236 (PMC6160749; doi:10.3389/fmicb.2018.02236)
Supplement: Supplementary file 6 [file Image_2.PDF]

frontiers

Supplementary Material

Microbial changes and host response in F344 rat colon depending on sex and age following a high-fat diet

Sun Min Lee, Nayoung Kim*, Hyuk Yoon, Ryoung Hee Nam, Dong Ho Lee

*** Correspondence:** Nayoung Kim: nakim49@snu.ac.kr

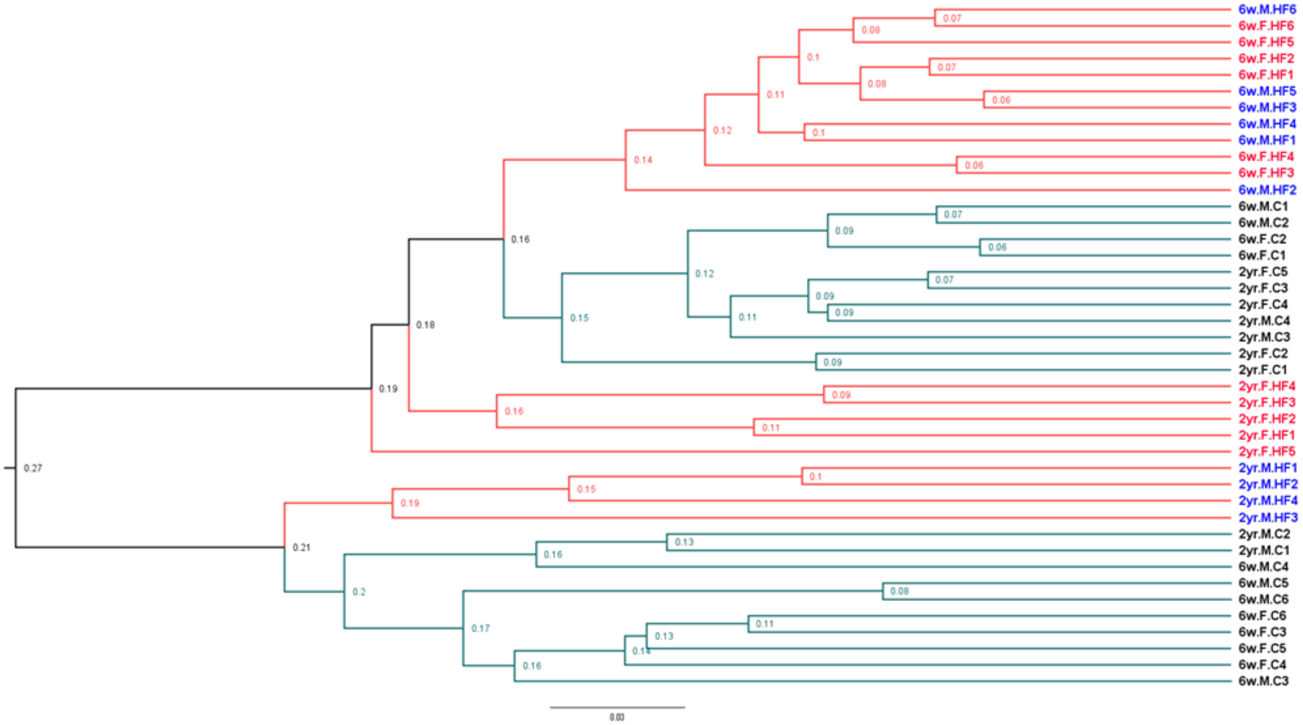

Supplementary Figure S2. Unweighted pair-group method with arithmetic mean (UPGMA) tree of Unifrac distances of the fecal samples; the green and red lines indicate the control and high-fat diet groups, respectively. Red and blue taxa characters indicate male and female, respectively. The UPGMA tree shows the clustering according to age, diet, and sex.
