# Supplementary material for: Microbial Changes and Host Response in F344 Rat Colon Depending on Sex and Age Following a High-Fat Diet
Source: Front Microbiol. 2018 Sep 21;9:2236. doi: 10.3389/fmicb.2018.02236 (PMC6160749; doi:10.3389/fmicb.2018.02236)
Supplement: Supplementary file 8 [file Image_4.pdf]

Supplementary Material

Microbial changes and host response in F344 rat colon depending on sex and age following a high-fat diet

Sun Min Lee, Nayoung Kim*, Hyuk Yoon, Ryoung Hee Nam, Dong Ho Lee

*** Correspondence:** Nayoung Kim: nakim49@snu.ac.kr

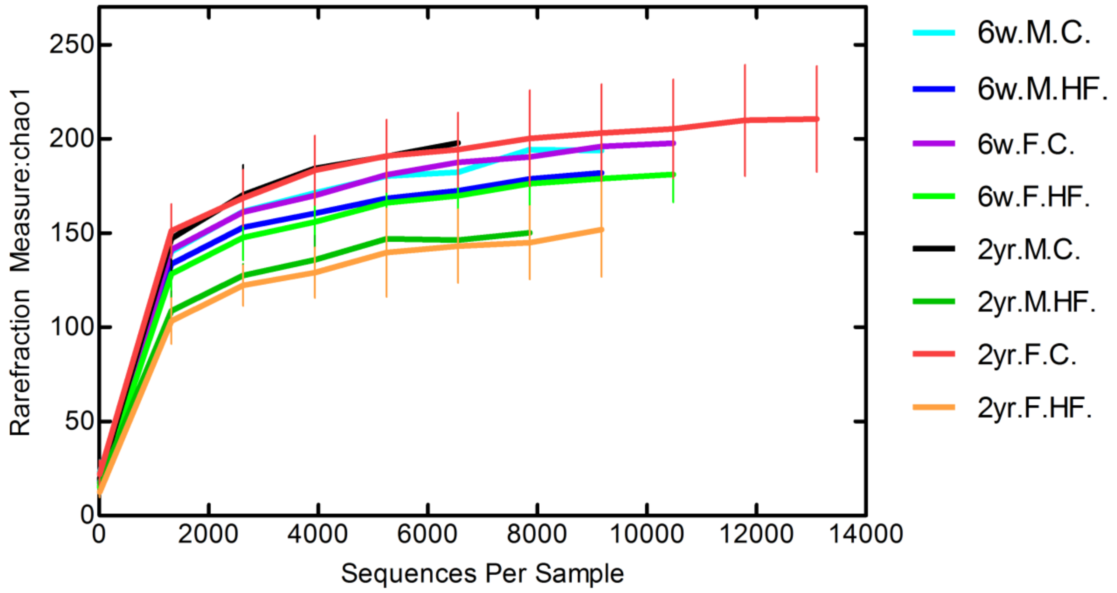

Supplementary Figure S4. Rarefaction curves of Chao1.
